# Stroke-associated pneumonia after acute ischemic stroke: early temporal dynamics, nonlinear risk factors, and time-dependent prognostic impact

**DOI:** 10.3389/fneur.2026.1841237

**Published:** 2026-10-20

**Authors:** Shitong Zhang, Qiaomei Yan, Zheng Wang, Rudong Xie, Ye Tao, Yingju Jin, Wendong Zuo

**Affiliations:** 1 Department of Neurology, Qianwei Hospital of Jilin Province, Changchun, Changchun, Jilin, China; 2 Department of Respiratory Medicine, Qianwei Hospital of Jilin Province, Changchun, Jilin, China; 3 Department of Thoracic Surgery, Qianwei Hospital of Jilin Province, Changchun, Changchun, Jilin, China; 4 Department of Rehabilitation Medicine, China-Japan Union Hospital of Jilin University, Changchun, Jilin, China

**Keywords:** acute ischemic stroke, in-hospital outcomes, nonlinear associations, risk factors, stroke-associated pneumonia

## Abstract

**Background:**

Stroke-associated pneumonia (SAP) is a common complication following acute ischemic stroke, yet its early occurrence patterns, nonlinear risk factors, and time-dependent prognostic impact remain incompletely characterized. We investigated SAP incidence, temporal distribution, risk factors, and independent impact on in-hospital outcomes.

**Methods:**

This single-center retrospective cohort study included 3,347 consecutive patients with acute ischemic stroke (2019–2025). SAP was diagnosed within 7 days after admission using consensus criteria. Cox models with restricted cubic splines identified risk factors and nonlinear relationships. Time-dependent Cox models, Gamma generalized linear models, and logistic regression were used to assess associations between SAP and in-hospital outcomes. Multiple imputation, complete-case, and landmark analyses verified robustness.

**Results:**

SAP developed in 428 patients (12.8%), with 55.1% occurring within 72 h (peak day 3). Independent risk factors included older age (HR 1.02), pre-stroke mRS 3–5 (HR 1.39), COPD (HR 1.69), cardioembolism (HR 1.71), dysphagia (HR 2.02), and nasogastric tube insertion within 48 h (HR 2.31) (all *p* < 0.05). Admission NIHSS, neutrophil-lymphocyte ratio, and albumin showed nonlinear associations with SAP risk (all P-nonlinear <0.05). Time-dependent analyses revealed SAP independently associated with in-hospital death (HR 2.34), ICU transfer (HR 2.81), invasive mechanical ventilation (HR 3.47), prolonged length of stay (RoM 1.35), and poor functional outcome (OR 1.90) (all *p* < 0.001). Sensitivity analyses confirmed robustness.

**Conclusion:**

SAP occurs early after acute ischemic stroke, with identifiable risk factors exhibiting nonlinear relationships, and independently predicts adverse in-hospital outcomes. Findings support forward-shifted preventive strategies, including early swallowing assessment, feeding pathway optimization, airway protection, and nutritional support, in high-risk patients during the first days of hospitalization.

## 1 Introduction

Stroke remains a leading cause of mortality and disability worldwide, with China bearing a particularly substantial burden. In 2021, 4.09 million new stroke cases and 2.59 million stroke-related deaths occurred in China, with ischemic stroke accounting for the majority (National Program for Stroke Prevention and Million Disability Reduction Initiative Expert Committee, 2025). As population aging accelerates and the number of stroke survivors rises, (1) contemporary management of acute ischemic stroke (AIS) has evolved beyond reperfusion therapy alone, with increasing recognition that complication control directly influences short-term outcomes (2).

Among post-stroke complications, stroke-associated pneumonia (SAP) represents the most common and clinically consequential infectious complication (3). SAP prolongs hospitalization, increases healthcare costs, and independently contributes to poorer functional recovery and elevated mortality. A systematic review estimated the pooled incidence of post-stroke pneumonia at approximately 14.3%, although considerable heterogeneity across studies, largely attributable to inconsistent diagnostic criteria, remains (4).

The temporal profile of SAP is notably predictable. De Jonge et al. (5) demonstrated that pneumonia predominantly manifests within the first week following stroke onset, peaking on day 3. This early clustering carries important implications for clinical surveillance and the timing of preventive interventions. The pathogenesis of SAP is multifactorial, operating through intersecting mechanisms: aspiration due to dysphagia and impaired cough reflex, stroke-induced immunodepression (6), decreased airway clearance, and underlying patient frailty (7). Dysphagia serves as a critical gateway to aspiration pneumonia, with risk amplified by neurological impairment severity and specific lesion locations (8, 9).

Substantial research has identified patients at elevated risk of SAP. Systematic reviews consistently report advanced age, stroke severity, chronic obstructive pulmonary disease, dysphagia, and nasogastric tube insertion as key determinants (10, 11). Building on these associations, several clinical risk scores, including A2DS2, ISAN, and PANTHERIS, have been developed for early risk stratification (12).

Despite these advances, critical gaps persist. First, considerable population heterogeneity remains problematic. Many studies have pooled ischemic stroke and intracerebral hemorrhage despite their distinct pathophysiological profiles (13), potentially obscuring risk factors specific to AIS. Second, the relationship between SAP and outcomes has often been examined using approaches vulnerable to time-related bias. SAP is inherently a time-dependent exposure, occurring at varying time points during hospitalization rather than being present at admission. Treating SAP as a fixed baseline covariate introduces immortal time bias and may overestimate its true effect (14). This methodological concern has received insufficient attention, potentially compromising previous effect estimates. Third, the literature has predominantly focused on risk prediction or prognostic impact in isolation, with few studies integrating both. Whether SAP independently alters in-hospital trajectories or merely serves as a marker of severe stroke remains incompletely resolved (15, 16). Fourth, the functional form of continuous predictors has been largely overlooked. Most studies assume linear relationships between continuous variables (e.g., NIHSS score, inflammatory markers, nutritional indicators) and SAP risk, an assumption that may not reflect biological reality. Emerging evidence suggests that biomarkers such as the neutrophil-to-lymphocyte ratio (NLR) and serum albumin exhibit nonlinear associations with post-stroke complications (17), yet this complexity is rarely incorporated into prediction frameworks.

To address these gaps, we conducted a large retrospective cohort study focusing exclusively on AIS patients. Our investigation had three objectives. First, we characterized SAP timing and burden using standardized, consensus-based diagnostic criteria (18), providing contemporary incidence estimates and clarifying the distribution of early events. Second, we comprehensively identified factors independently associated with SAP, employing restricted cubic splines to model nonlinear relationships for continuous variables. This permits more accurate risk characterization and identification of potential intervention thresholds. Third, we quantified SAP’s independent impact on in-hospital outcomes, including mortality, intensive care unit transfer, invasive mechanical ventilation, length of stay, and discharge functional status, using time-dependent Cox models to eliminate immortal time bias and provide valid effect estimates.

By integrating high-risk identification and time-dependent prognostic assessment within a single analytical framework, this study aims to generate clinically actionable evidence to inform early risk stratification and targeted preventive strategies. Understanding not only who is at risk but also how SAP independently shapes hospital trajectories is essential for optimizing acute stroke care and improving patient outcomes (19, 20). Our findings have the potential to refine current management paradigms by emphasizing forward-shifted preventive interventions, including early swallowing assessment, judicious selection of feeding pathways, airway protection, and nutritional optimization, in high-risk AIS patients during the critical first days of hospitalization.

## 2 Materials and methods

### 2.1 Study design and setting

This single-center retrospective cohort study was conducted at the Department of Neurology at Qianwei Hospital of Jilin Province, Changchun City, China, and included patients admitted between January 1, 2019, and June 30, 2025. Data were collected from multiple hospital information systems: the electronic medical record system, the medical record front-page information system, the laboratory information system, the picture archiving and communication system, and the nursing record system. The admission date of the initial hospitalization served as time zero, with follow-up continuing until discharge or in-hospital death.

The study design, conduct, and reporting adhered to the Strengthening the Reporting of Observational Studies in Epidemiology (STROBE) statement guidelines. The study protocol was approved by the hospital ethics committee of Qianwei Hospital of Jilin (approval No. 2025101006), and informed consent was waived due to the study’s retrospective nature. All data were de-identified after extraction, and the principles of the Declaration of Helsinki were followed throughout.

### 2.2 Study population and cohort construction

We identified all hospitalized patients with a discharge diagnosis of acute ischemic stroke during the study period using International Classification of Diseases, Tenth Revision (ICD-10) codes from the medical record front page. Each case was subsequently verified through independent review of medical course records and neuroimaging data. Acute ischemic stroke was defined as the presence of acute focal neurological deficits consistent with brain infarction lesions confirmed by cranial magnetic resonance imaging, diffusion-weighted imaging, or cranial computed tomography during hospitalization. Onset time was determined from medical records, with wake-up stroke defined using the last known well time.

#### 2.2.1 Inclusion criteria

Patients were eligible for inclusion if they met all of the following criteria: age ≥18 years; onset-to-admission time ≤7 days; and completion of the index hospitalization with the Department of Neurology as the primary admitting service.

#### 2.2.2 Exclusion criteria

Patients were excluded from the analysis if they met any of the following criteria: final diagnosis of transient ischemic attack, intracranial hemorrhagic stroke (intracerebral hemorrhage or subarachnoid hemorrhage), or cerebral venous thrombosis; evidence of lower respiratory tract infection or pneumonia already present at admission (defined as chest imaging within 24 h showing infectious infiltrates or consolidation, with anti-infective treatment initiated on the first hospital day and pneumonia documented as the primary diagnosis); presence of any active infection requiring systemic anti-infective treatment at the time of admission; transfer from another hospital with a hospitalization duration exceeding 48 h prior to transfer; or missing data for the primary outcome (in-hospital death). For patients with multiple hospitalizations during the study period, only the first eligible index hospitalization was included to ensure statistical independence of observations.

### 2.3 Definition and adjudication of stroke-associated pneumonia

Stroke-associated pneumonia (SAP) was defined as new-onset pneumonia occurring within 7 days of admission during the index hospitalization, after excluding pneumonia present at admission, according to established definitions (15). Ventilator-associated pneumonia (VAP) was not analyzed separately; patients who developed pneumonia after endotracheal intubation were classified as having SAP if they met the PISC criteria, as our study aimed to capture all early-onset pneumonias following acute ischemic stroke. Chest imaging was performed at admission for all patients as part of routine stroke workup to exclude pneumonia present at admission. During hospitalization, chest X-ray or CT was performed when clinical symptoms suggestive of pneumonia developed (e.g., fever, cough, purulent sputum, oxygen desaturation). SAP diagnosis required new or progressive infiltrates on imaging performed after symptom onset. The diagnosis required all three of the following criteria to be met simultaneously, based on the Pneumonia in Stroke Consensus (PISC) Group recommendations (18), which are specifically designed for stroke-associated pneumonia and differ from the CDC or modified CDC surveillance definitions for general hospital-acquired pneumonia: (1) new or progressive infiltrates or consolidation on chest X-ray or chest computed tomography; (2) at least one systemic inflammatory response criterion (body temperature ≥38.0 °C or ≤36.0 °C; white blood cell count >10 × 10^9^/L or <4 × 10^9^/L); and (3) at least one respiratory manifestation (new cough; purulent sputum; rales on auscultation; room air oxygen saturation ≤90% or increased oxygen requirement compared with the previous day). The date of SAP occurrence was defined as the first time all conditions were simultaneously satisfied.

SAP adjudication was performed independently by two researchers who were blinded to outcome information. In cases of disagreement, a third senior physician reviewed the case and rendered a final decision.

### 2.4 Definitions and adjudication of in-hospital outcomes

The primary outcome was in-hospital all-cause death, determined from discharge records. Secondary outcomes included: (1) intensive care unit (ICU) transfer, defined as transfer from a general ward to the ICU during the index hospitalization; (2) invasive mechanical ventilation, defined as endotracheal intubation with subsequent mechanical ventilation; (3) length of hospital stay, counted as calendar days from admission to discharge (for patients who died in hospital, from admission to death); and (4) functional outcome at discharge, assessed using the modified Rankin Scale (mRS), with scores of 0–2 defined as good functional outcome and scores of 3–6 as poor functional outcome (in-hospital death recorded as mRS 6) (21).

### 2.5 Collection and operational definitions of candidate risk factors and covariates

Baseline characteristics and clinical variables were extracted by researchers blinded to outcomes, with the collection window uniformly limited to the first assessment or laboratory result completed within 24 h after admission. Demographic variables included age and sex. Pre-stroke functional status was determined from the admission history using the pre-stroke mRS, stratified as 0–2 versus 3–5. Comorbidities were ascertained from both past medical history and discharge diagnoses, including hypertension, diabetes mellitus, atrial fibrillation, coronary heart disease, chronic heart failure, chronic obstructive pulmonary disease, chronic kidney disease, and previous stroke.

Stroke severity was assessed using the first National Institutes of Health Stroke Scale (NIHSS) score within 24 h after admission. Consciousness level was evaluated using the Glasgow Coma Scale (GCS) score at admission. Stroke subtype was classified according to the Trial of Org 10172 in Acute Stroke Treatment (TOAST) criteria recorded after discharge. Infarct vascular territory was determined from imaging reports, with posterior circulation infarction defined as involvement of the vertebrobasilar arterial supply territory. All patients received standard acute ischemic stroke management according to current guidelines, including intravenous thrombolysis within 4.5 h and mechanical thrombectomy within 24 h when indicated. Non-SAP complications were managed according to institutional protocols, including blood pressure control, glycemic management, fever control, and prevention of deep vein thrombosis. Swallowing function was assessed using the Kubota water-drinking test (grades 1–5, with grade ≥3 defined as dysphagia), completed within 24 h of admission. Nasogastric tube insertion was determined by the treating physicians based on standardized dysphagia screening (Kubota grade ≥3) combined with clinical evaluation of stroke severity (e.g., admission NIHSS ≥16), impaired consciousness (GCS < 12), or clinically evident aspiration risk, reflecting real-world clinical practice. SAP was managed according to institutional protocols based on current guidelines, including empiric antibiotic therapy initiated after diagnosis, respiratory support as needed, and supportive care.

Early in-hospital management variables included intravenous thrombolysis, mechanical thrombectomy, and nasogastric tube insertion within 48 h after admission. Antibiotic use was not recorded as a structured variable because antibiotics were initiated after SAP diagnosis at the discretion of the treating physicians and were documented in narrative form; therefore, antibiotic use was not included as a covariate to avoid overadjustment bias, as it lies on the causal pathway between SAP and outcomes. Laboratory variables were obtained from the first blood tests within 24 h after admission. They included white blood cell count, absolute neutrophil count, absolute lymphocyte count, serum albumin, blood glucose, and serum creatinine. The neutrophil-to-lymphocyte ratio (NLR) was calculated from absolute counts.

### 2.6 Data extraction, database construction, and quality control

A structured database was established according to a predefined data dictionary, with independent double data entry and verification performed by two researchers. Temporal consistency checks were conducted for all key time variables (admission date, SAP occurrence date, ICU transfer date, mechanical ventilation start date, and discharge or death date). Continuous values outside physiological ranges were verified by tracing back to the original laboratory reports. All imaging and clinical record information required for SAP and outcome adjudication was extracted directly from sources. Following database completion, all study identifiers were de-identified, retaining only necessary analysis fields, and access permissions were restricted.

### 2.7 Statistical analysis

*Descriptive statistics*: Continuous variable normality was assessed using the Shapiro–Wilk test and distribution plots. Normally distributed variables were presented as mean ± standard deviation and compared using the independent-samples *t*-test; non-normally distributed variables were presented as median (interquartile range) and compared using the Mann–Whitney U test. Categorical variables were presented as counts (percentages) and compared using the χ^2^ test or Fisher’s exact test, as appropriate.

#### 2.7.1 Risk factor analysis for SAP

Using the admission date as time zero, patients were followed until SAP occurrence, discharge, or in-hospital death, with a maximum follow-up of 7 days. Cox proportional hazards models estimated hazard ratios (HRs) and 95% confidence intervals (CIs) for candidate variables associated with SAP occurrence. Variables with *p* < 0.10 in univariable analysis were entered into the multivariable model, with age, sex, and admission NIHSS score forced into the model regardless of univariable significance. Continuous variables were initially entered in their original form, and restricted cubic splines with three knots at the 10th, 50th, and 90th percentiles were used to test the linearity assumption. Variables violating the linearity assumption were modeled using splines. Multicollinearity was assessed using the variance inflation factor, with a threshold of >5 indicating problematic collinearity; when present, the variable with stronger clinical significance was retained. The proportional hazards assumption was tested using Schoenfeld residuals.

#### 2.7.2 Prognostic impact analysis of SAP

To control for time-related bias, the association between SAP and in-hospital outcomes was analyzed using a time-dependent exposure approach. For the primary outcome of in-hospital death, a time-dependent Cox model was constructed with admission date as time zero and hospital survivors censored at discharge. SAP was treated as a time-dependent covariate, assigned a value of 0 before SAP occurrence and 1 thereafter. Adjustment variables were prespecified based on clinical relevance. They included: age, sex, pre-stroke mRS stratification, admission NIHSS, admission GCS, dysphagia, posterior circulation infarction, TOAST subtype, atrial fibrillation, diabetes mellitus, chronic obstructive pulmonary disease, albumin, blood glucose, intravenous thrombolysis, mechanical thrombectomy, and nasogastric tube insertion within 48 h after admission.

For secondary time-to-event outcomes (ICU transfer and invasive mechanical ventilation), identical time-dependent Cox models were used, with patients censored at discharge or death. The length of hospital stay was analyzed among hospital survivors using a Gamma generalized linear model with a log link, adjusting for the same covariates. Functional outcome at discharge (poor outcome defined as mRS 3–5) was analyzed among hospital survivors using binary logistic regression, with identical covariate adjustment.

#### 2.7.3 Handling of missing data

Variables with missing rates ≤20% were imputed using multiple imputation by chained equations, generating 10 imputed datasets. Continuous variables were imputed using predictive mean matching, binary variables using logistic regression, and ordered categorical variables using proportional odds models. The imputation model included all covariates and study outcome variables. Regression results from imputed datasets were combined using Rubin’s rules.

#### 2.7.4 Sensitivity analyses

Two sensitivity analyses were performed to assess result robustness: (1) a complete-case analysis repeating all primary analyses using only cases with complete data; and (2) a day-3 landmark analysis, including only patients hospitalized for at least 3 days, defining SAP occurring within the first 3 days as a fixed exposure, and commencing follow-up from day 3 onward.

All tests were two-sided, with *p* < 0.05 considered statistically significant. Statistical analyses were performed using R software version 4.3.2 (R Foundation for Statistical Computing, Vienna, Austria).

## 3 Results

### 3.1 Study population screening and cohort construction

A total of 3,347 hospitalized patients with acute ischemic stroke were included in the final analysis. Among these, 428 patients (12.8%) developed stroke-associated pneumonia (SAP) during hospitalization, while 2,919 patients (87.2%) did not experience SAP. The flow diagram of patient selection and exclusion is shown in Figure 1.

**Figure 1:**
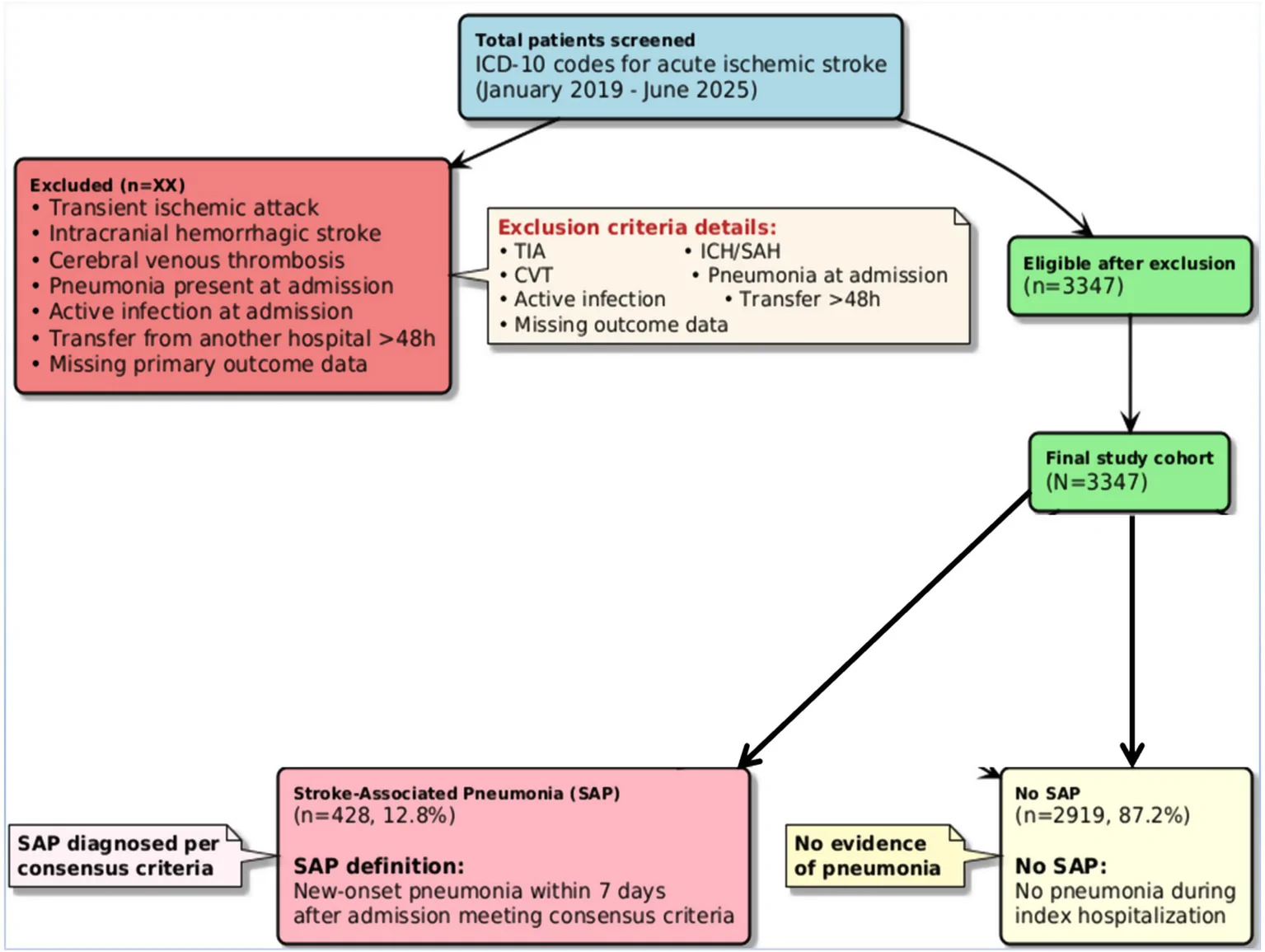
Flowchart of study population screening and cohort construction.

### 3.2 Baseline characteristics and data completeness

Table 1 presents the baseline characteristics of the study cohort stratified by SAP status. Compared with patients who did not develop SAP, those in the SAP group were significantly older and exhibited poorer pre-stroke functional status, with a higher proportion having pre-stroke modified Rankin Scale scores of 3–5 (22.43% vs. 8.15%, *p* < 0.001). The prevalence of multiple comorbidities was substantially higher in the SAP group, including hypertension, diabetes mellitus, atrial fibrillation, coronary heart disease, chronic heart failure, chronic obstructive pulmonary disease, chronic kidney disease, and previous stroke (all *p* < 0.05).

**Table 1:** Baseline characteristics of the study cohort stratified by stroke-associated *pneumonia* status.

Variable	Overall (*N* = 3,347)	No SAP (*n* = 2,919)	SAP (*n* = 428)	*p*-value
A. Demographics and pre-stroke status				
Age, years, mean ± SD	67.84 ± 11.59	66.92 ± 11.48	74.11 ± 10.37	<0.001
Male, *n* (%)	(2114). (63.16)	1828 (62.62)	286 (66.82)	0.093
Pre-stroke mRS score 3–5, *n* (%)	334 (9.98)	238 (8.15)	96 (22.43)	<0.001
B. Comorbidities, *n* (%)				
Hypertension	2,316 (69.20)	1998 (68.45)	318 (74.30)	0.014
Diabetes mellitus	953 (28.47)	812 (27.82)	141 (32.94)	0.028
Atrial fibrillation	508 (15.18)	392 (13.43)	116 (27.10)	<0.001
Coronary heart disease	524 (15.66)	436 (14.94)	88 (20.56)	0.003
Chronic heart failure	145 (4.33)	103 (3.53)	42 (9.81)	<0.001
Chronic obstructive pulmonary disease	159 (4.75)	112 (3.84)	47 (10.98)	<0.001
Chronic kidney disease	190 (5.68)	151 (5.17)	39 (9.11)	0.001
Previous stroke	740 (22.11)	623 (21.34)	117 (27.34)	0.005
C. Stroke characteristics and neurological status				
Admission NIHSS score, median (IQR)	4 (2–8)	4 (1–7)	11 (6–16)	<0.001
Admission GCS score, median (IQR)	15 (15–15)	15 (15–15)	14 (12–15)	<0.001
TOAST subtype, *n* (%)				<0.001
Large-artery atherosclerosis	1,121 (33.49)	989 (33.88)	132 (30.84)	
Cardioembolism	693 (20.71)	544 (18.64)	149 (34.81)	
Small-artery occlusion	820 (24.50)	779 (26.69)	41 (9.58)	
Other determined etiology	121 (3.62)	104 (3.56)	17 (3.97)	
Undetermined etiology	592 (17.69)	503 (17.23)	89 (20.79)	
Posterior circulation infarction, *n* (%)	579 (17.30)	481 (16.48)	98 (22.90)	0.001
Dysphagia, *n* (%)	578 (17.27)	347 (11.89)	231 (53.97)	<0.001
D. Early in-hospital treatment and management, *n* (%)				
Intravenous thrombolysis	386 (11.53)	328 (11.24)	58 (13.55)	0.161
Mechanical thrombectomy	166 (4.96)	127 (4.35)	39 (9.11)	<0.001
Nasogastric tube insertion within 48 h	362 (10.82)	189 (6.47)	173 (40.42)	<0.001
E. Laboratory indicators within 24 h after admission				
White blood cell count, ×10^9^/L, median (IQR)	7.26 (6.02–9.07)	7.11 (5.94–8.79)	8.83 (7.26–11.13)	<0.001
Absolute neutrophil count, ×10^9^/L, median (IQR)	4.96 (3.87–6.81)	4.81 (3.75–6.52)	6.79 (5.17–8.99)	<0.001
Absolute lymphocyte count, ×10^9^/L, median (IQR)	1.60 (1.24–2.02)	1.66 (1.29–2.07)	1.22 (0.91–1.59)	<0.001
Neutrophil-to-lymphocyte ratio, median (IQR)	3.05 (2.09–4.76)	2.88 (1.99–4.37)	5.27 (3.56–7.93)	<0.001
Serum albumin, g/L, mean ± SD	39.71 ± 4.29	40.08 ± 4.07	37.16 ± 4.83	<0.001
Blood glucose, mmol/L, median (IQR)	6.24 (5.43–7.51)	6.16 (5.37–7.33)	7.11 (6.00–8.84)	<0.001
Serum creatinine, μmol/L, median (IQR)	73.4 (61.8–88.7)	72.7 (61.3–87.5)	79.5 (65.1–96.6)	0.002

Data are presented as mean ± SD, median (IQR), or *n* (%). *p*-values from *t*-test, Mann–Whitney U test, χ^2^ test, or Fisher’s exact test. SAP, stroke-associated pneumonia; SD, standard deviation; IQR, interquartile range; mRS, modified Rankin Scale; NIHSS, National Institutes of Health Stroke Scale; GCS, Glasgow Coma Scale; TOAST, Trial of Org 10172 in Acute Stroke Treatment.

Regarding stroke characteristics, patients with SAP presented with greater neurological deficit severity, as reflected by higher median admission National Institutes of Health Stroke Scale scores (11 [interquartile range {IQR} 6–16] vs. 4 [IQR 1–7], *p* < 0.001) and lower median Glasgow Coma Scale scores (14 [IQR 12–15] vs. 15 [IQR 15–15], *p* < 0.001). The distribution of TOAST subtypes differed significantly between groups (*p* < 0.001), with cardioembolism being more frequent in the SAP group (34.81% vs. 18.64%). Posterior circulation infarction was also more common among SAP patients (22.90% vs. 16.48%, *p* = 0.001). Dysphagia was present in over half of SAP patients (53.97%) compared with only 11.89% of those without SAP (*p* < 0.001).

Early in-hospital management differed between groups, with higher rates of mechanical thrombectomy (9.11% vs. 4.35%, *p* < 0.001) and nasogastric tube insertion within 48 h (40.42% vs. 6.47%, *p* < 0.001) observed in the SAP group. Laboratory investigations revealed a more pronounced inflammatory profile in SAP patients, characterized by higher white blood cell counts, absolute neutrophil counts, and neutrophil-to-lymphocyte ratios, together with lower absolute lymphocyte counts and serum albumin levels (all *p* < 0.001). Blood glucose and serum creatinine concentrations were also significantly elevated in the SAP group.

Data completeness for covariates included in the regression models was high, as shown in Table 2. The majority of variables had no missing values. Among those with missing data, serum albumin exhibited the highest missing rate (2.12%, 71 patients), followed by admission Glasgow Coma Scale score (1.76%, 59 patients) and admission National Institutes of Health Stroke Scale score (1.37%, 46 patients). All other variables had missing rates below 1.1%. Crucially, there were no missing values for any of the study outcome variables.

**Table 2:** Missing data for covariates included in regression models.

Variable	Models in which the variable was entered	Missing values, *n* (%)
Admission NIHSS score	Risk factor and outcome models	46 (1.37)
Admission GCS score	Risk factor and outcome models	59 (1.76)
White blood cell count	SAP risk factor model only	19 (0.57)
Absolute neutrophil count	SAP risk factor model only	28 (0.84)
Absolute lymphocyte count	SAP risk factor model only	33 (0.99)
Neutrophil-to-lymphocyte ratio	SAP risk factor model only	34 (1.02)
Serum albumin	Risk factor and outcome models	71 (2.12)
Blood glucose	Risk factor and outcome models	23 (0.69)
Serum creatinine	SAP risk factor model only	17 (0.51)

Only covariates with missing values entered in regression models are shown. All other covariates had complete data. Multiple imputation by chained equations generated 10 imputed datasets. No outcome variables had missing values. NIHSS, National Institutes of Health Stroke Scale; GCS, Glasgow Coma Scale; SAP, stroke-associated pneumonia.

### 3.3 Occurrence characteristics of stroke-associated pneumonia

Among the 428 SAP events, the majority occurred early during hospitalization. More than half of all SAP cases (55.14%) developed within the first 72 h after admission, with the highest incidence observed on day 3, accounting for 25.47% of all events. Thereafter, the number of new cases declined progressively, with only 13.32% of SAP events occurring on days 6 and 7 combined. The temporal distribution of SAP onset is illustrated in Figure 2.

**Figure 2:**
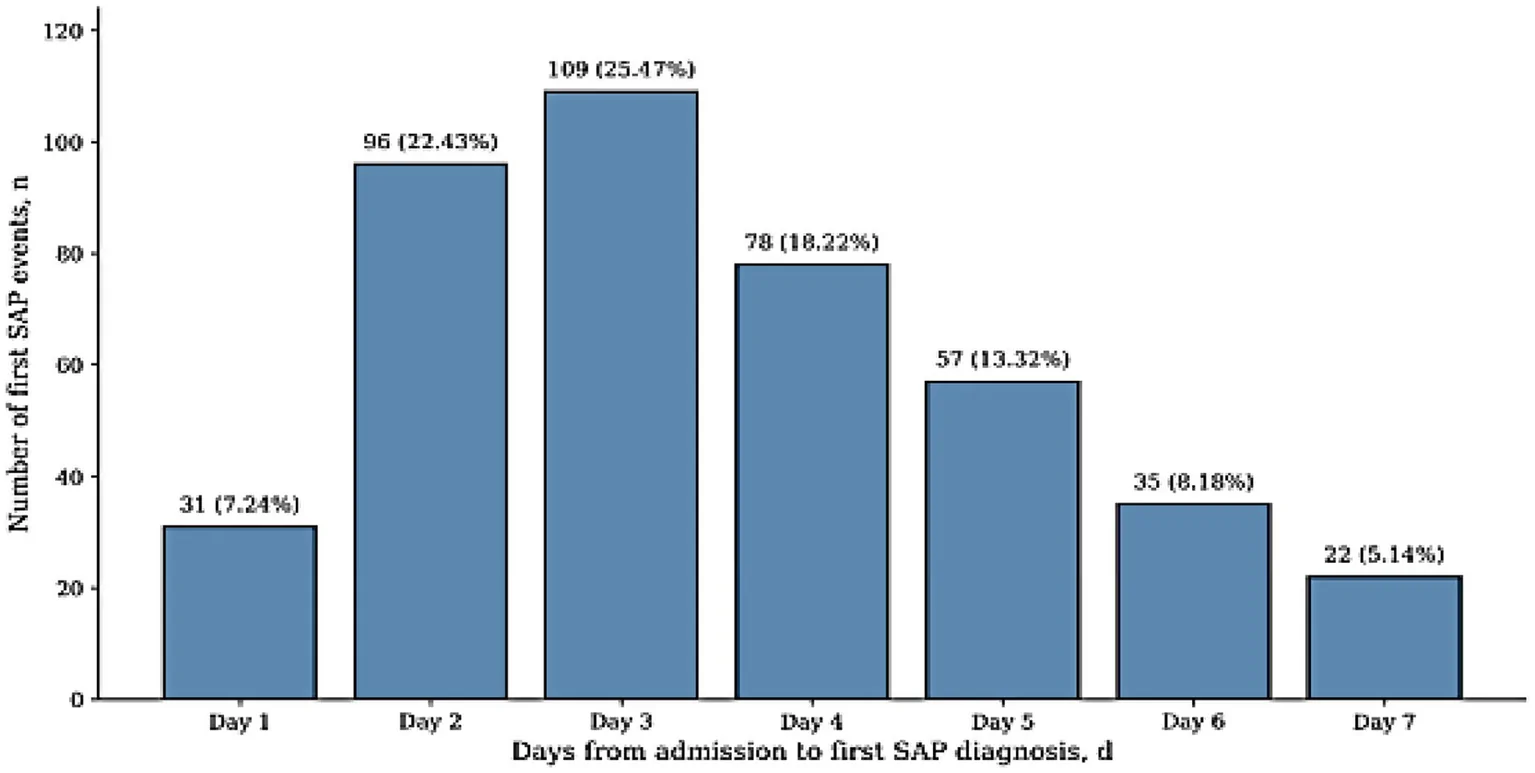
Temporal distribution of stroke-associated pneumonia onset within 7 days after admission. Day 1 was defined as 0–24 h after admission. The figure displays only the first SAP events occurring within 7 days after admission (*n* = 428); patients without SAP are not shown.

### 3.4 Risk factors for stroke-associated pneumonia

Table 3 presents the results of univariable and multivariable Cox regression analyses for factors associated with SAP occurrence. In the multivariable model, several factors demonstrated independent associations with SAP risk. Older age (hazard ratio [HR] 1.02 per year, 95% confidence interval [CI] 1.01–1.03, *p* < 0.001), pre-stroke mRS score 3–5 (HR 1.39, 95% CI 1.06–1.81, *p* = 0.016), and chronic obstructive pulmonary disease (HR 1.69, 95% CI 1.22–2.34, *p* = 0.002) were independently associated with increased SAP risk. Among stroke subtypes, cardioembolism remained independently associated with SAP (HR 1.71, 95% CI 1.16–2.52, *p* = 0.007) compared with small-artery occlusion as the reference category. Dysphagia (HR 2.02, 95% CI 1.59–2.57, *p* < 0.001) and nasogastric tube insertion within 48 h after admission (HR 2.31, 95% CI 1.80–2.97, *p* < 0.001) exhibited the strongest independent associations with SAP. Because nasogastric tube insertion is a clinical decision driven by dysphagia and stroke severity, this association should be interpreted as non-causal; nasogastric tube insertion likely serves as both a marker of severe illness and a potential mediator of aspiration risk.

**Table 3:** Cox regression analysis of factors associated with stroke-associated pneumonia.

Variable	Univariable	Multivariable	HR (95% CI)	*p*-value
	HR (95% CI)	*p*-value		
Panel A: Linear terms and categorical variables				
Age, years	1.04 (1.03–1.05)	<0.001	1.02 (1.01–1.03)	<0.001
Male	1.18 (0.97–1.43)	0.095	1.10 (0.89–1.36)	0.381
Pre-stroke mRS score 3–5	2.86 (2.25–3.62)	<0.001	1.39 (1.06–1.81)	0.016
Hypertension	1.28 (1.03–1.58)	0.023	1.07 (0.85–1.34)	0.567
Diabetes mellitus	1.24 (1.01–1.50)	0.036	1.09 (0.88–1.35)	0.434
Atrial fibrillation	2.08 (1.70–2.56)	<0.001	1.18 (0.90–1.53)	0.228
Coronary heart disease	1.41 (1.13–1.76)	0.003	1.00 (0.77–1.30)	0.986
Chronic heart failure	2.41 (1.78–3.27)	<0.001	1.21 (0.85–1.72)	0.289
Chronic obstructive pulmonary disease	2.74 (2.05–3.66)	<0.001	1.69 (1.22–2.34)	0.002
Chronic kidney disease	1.67 (1.23–2.28)	0.001	1.09 (0.77–1.54)	0.635
Previous stroke	1.32 (1.07–1.63)	0.010	1.04 (0.83–1.31)	0.724
Admission GCS score, points	0.79 (0.75–0.83)	<0.001	0.97 (0.91–1.02)	0.243
TOAST subtype (reference: small-artery occlusion)				
Large-artery atherosclerosis	2.43 (1.73–3.42)	<0.001	1.24 (0.85–1.80)	0.268
Cardioembolism	4.46 (3.19–6.23)	<0.001	1.71 (1.16–2.52)	0.007
Other determined etiology	2.83 (1.52–5.27)	0.001	1.35 (0.69–2.64)	0.383
Undetermined etiology	3.08 (2.15–4.42)	<0.001	1.19 (0.80–1.77)	0.394
Posterior circulation infarction	1.44 (1.15–1.80)	0.001	1.12 (0.88–1.42)	0.366
Dysphagia	6.08 (4.93–7.50)	<0.001	2.02 (1.59–2.57)	<0.001
Intravenous thrombolysis	1.16 (0.92–1.46)	0.206	—	—
Mechanical thrombectomy	2.09 (1.50–2.90)	<0.001	1.16 (0.82–1.65)	0.399
Nasogastric tube insertion within 48 h	6.74 (5.43–8.35)	<0.001	2.31 (1.80–2.97)	<0.001
White blood cell count, ×10^9^/L	1.10 (1.08–1.13)	<0.001	—	—
Absolute neutrophil count, ×10^9^/L	1.09 (1.07–1.12)	<0.001	—	—
Absolute lymphocyte count, ×10^9^/L	0.62 (0.54–0.72)	<0.001	—	—
Blood glucose, mmol/L	1.07 (1.04–1.10)	<0.001	1.02 (0.99–1.05)	0.169
Serum creatinine, μmol/L	1.004 (1.002–1.006)	0.001	1.001 (0.999–1.004)	0.247
Panel B. Continuous variables modeled with restricted cubic splines			P-overall	P-nonlinear
Admission NIHSS score, points	1.13 (1.12–1.15)	<0.001	<0.001	0.004
Neutrophil-to-lymphocyte ratio	1.10 (1.08–1.11)	<0.001	<0.001	0.018
Serum albumin, g/L	0.88 (0.86–0.90)	<0.001	<0.001	0.006

Reference categories: female (sex); mRS 0–2 (pre-stroke mRS); small-artery occlusion (TOAST). Follow-up: admission to SAP, discharge, death, or day 7. Variables with *p* < 0.10 in univariable analysis were entered into the multivariable model; age, sex, and NIHSS were forced into the model. WBC, neutrophil, and lymphocyte counts were excluded due to collinearity with NLR. Panel B: P-overall tests total contribution; P-nonlinear tests departure from linearity. Abbreviations: HR, hazard ratio; CI, confidence interval; mRS, modified Rankin scale; GCS, Glasgow coma scale; TOAST, trial of org 10,172 in acute stroke treatment; NIHSS, National Institutes of Health stroke scale; NLR, neutrophil-to-lymphocyte ratio.

Three continuous variables demonstrated significant nonlinear associations with SAP risk after adjustment: admission NIHSS score, neutrophil-to-lymphocyte ratio (NLR), and serum albumin (all P-overall <0.001; all P-nonlinear <0.05). The shapes of these associations are depicted in Figure 3. Higher admission NIHSS scores were associated with progressively greater SAP risk, with the slope of risk acceleration becoming steeper at higher NIHSS scores. Similarly, higher NLR values were associated with elevated SAP risk, with the risk gradient intensifying at higher NLR levels. Conversely, lower serum albumin concentrations were associated with markedly increased SAP risk, with the relationship exhibiting a nonlinear pattern whereby risk escalated more sharply below approximately 35 g/L.

**Figure 3:**
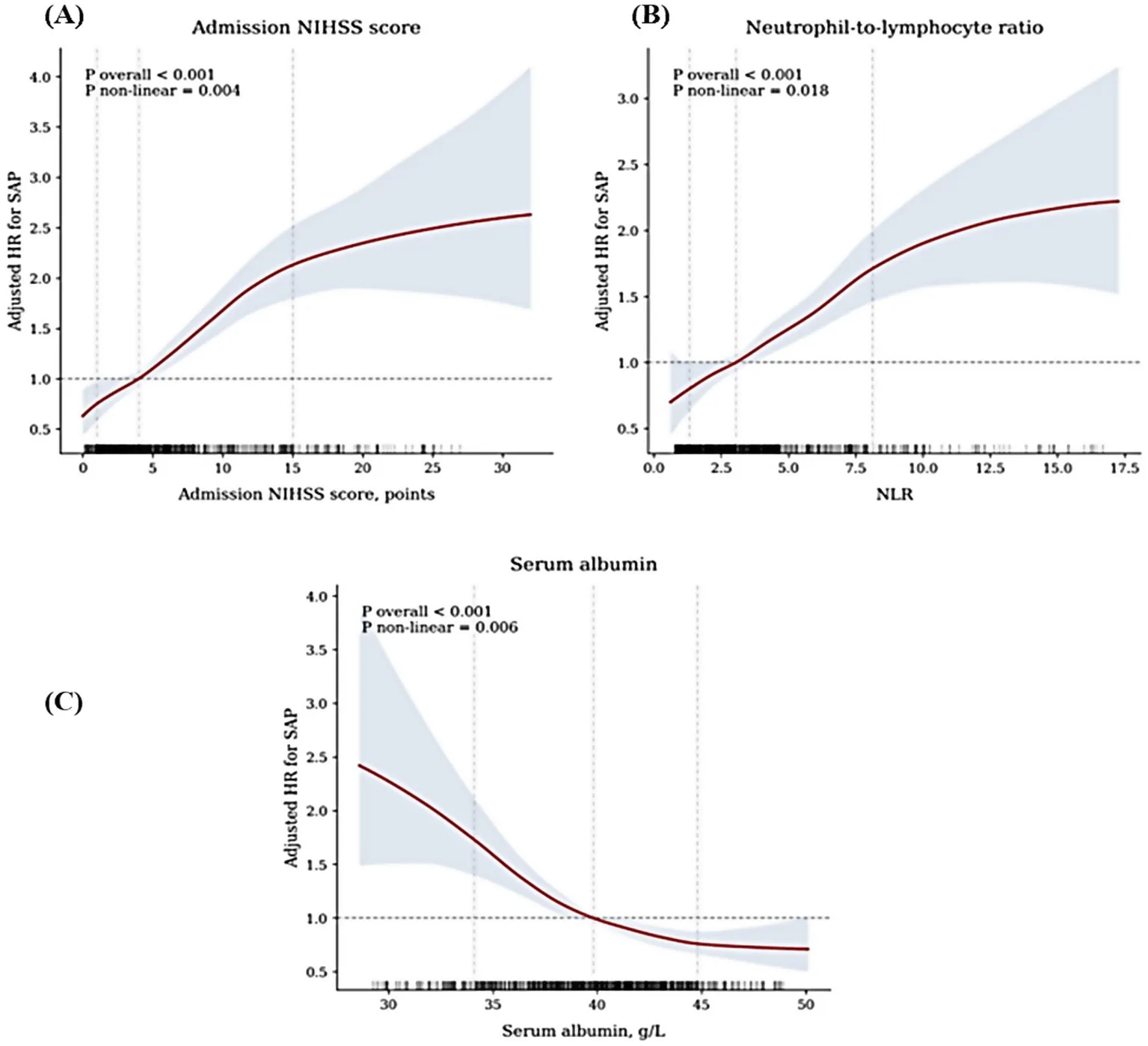
Restricted cubic spline plots illustrating nonlinear associations between continuous variables and stroke-associated pneumonia risk. Each panel corresponds to a variable modeled with restricted cubic splines in the multivariable Cox regression (Panel **B** of Table 3). The solid line represents the adjusted hazard ratio, with the shaded area indicating the 95% confidence interval. Rug plots on the x-axis show the distribution of each variable in the study sample. Knots were placed at the 10th, 50th, and 90th percentiles, with the median value serving as the reference (hazard ratio = 1.0). **(A)** Admission NIHSS score. **(B)** Neutrophil-to-lymphocyte ratio. **(C)** Serum albumin.

### 3.5 Association of stroke-associated pneumonia with in-hospital outcomes

Table 4 presents the associations between SAP and in-hospital outcomes derived from time-dependent analyses. After comprehensive adjustment for potential confounders, SAP remained significantly associated with an elevated risk of all primary and secondary outcomes. For time-to-event outcomes in the overall cohort, SAP was independently associated with in-hospital all-cause death (adjusted HR 2.34, 95% CI 1.71–3.20, *p* < 0.001), ICU transfer (adjusted HR 2.81, 95% CI 2.14–3.69, *p* < 0.001), and invasive mechanical ventilation (adjusted HR 3.47, 95% CI 2.48–4.86, *p* < 0.001).

**Table 4:** Association of stroke-associated pneumonia with in-hospital outcomes.

Outcome	No SAP	SAP	Crude effect (95% CI)	*p*-value	Adjusted effect (95% CI)	*p*-value
Time-to-event outcomes in the overall cohort						
In-hospital death, *n*/*N* (%)	112/2919 (3.84)	94/428 (21.96)	HR 4.68 (3.55–6.15)	<0.001	HR 2.34 (1.71–3.20)	<0.001
ICU transfer, *n*/*N* (%)	161/2919 (5.52)	136/428 (31.78)	HR 5.59 (4.47–7.00)	<0.001	HR 2.81 (2.14–3.69)	<0.001
Invasive mechanical ventilation, *n*/*N* (%)	72/2919 (2.47)	105/428 (24.53)	HR 7.63 (5.69–10.24)	<0.001	HR 3.47 (2.48–4.86)	<0.001
Outcomes among hospital survivors						
Length of hospital stay, days, median (IQR)	9 (7–12)	17 (13–24)	RoM 1.82 (1.73–1.92)	<0.001	RoM 1.35 (1.27–1.44)	<0.001
Poor outcome at discharge (mRS 3–5), *n*/*N* (%)	889/2807 (31.67)	214/334 (64.07)	OR 3.85 (3.01–4.94)	<0.001	OR 1.90 (1.43–2.52)	<0.001

Time-to-event outcomes: Cox models with SAP as time-dependent covariate. Length of stay: Gamma GLM with log link (ratio of means). Functional outcome: binary logistic regression (odds ratio). All models adjusted for: age, sex, pre-stroke mRS, NIHSS, GCS, dysphagia, posterior circulation infarction, TOAST subtype, atrial fibrillation, diabetes, COPD, albumin, glucose, thrombolysis, thrombectomy, and nasogastric tube insertion. SAP, stroke-associated pneumonia; CI, confidence interval; HR, hazard ratio; ICU, intensive care unit; IQR, interquartile range; RoM, ratio of means; OR, odds ratio; mRS, modified Rankin Scale.

Among patients who survived to hospital discharge, SAP remained independently associated with prolonged hospitalization and poorer functional recovery. The adjusted ratio of means for length of hospital stay was 1.35 (95% CI 1.27–1.44, *p* < 0.001), indicating that SAP was associated with a 35% relative increase in hospitalization duration. Furthermore, SAP was independently associated with nearly twofold higher odds of poor functional outcome (modified Rankin Scale score 3–5) at discharge (adjusted odds ratio 1.90, 95% CI 1.43–2.52, *p* < 0.001).

### 3.6 Sensitivity analyses

The robustness of the primary findings was evaluated through two complementary sensitivity analyses, summarized in Table 5. Complete-case analysis, restricted to patients with complete data for all covariates (*n* = 3,236 for time-to-event outcomes; *n* = 3,038 for hospital-survival outcomes), yielded effect estimates that closely resembled those from the primary multiple imputation analysis. SAP remained significantly associated with increased risks of in-hospital death (adjusted HR 2.29, 95% CI 1.66–3.15, *p* < 0.001), ICU transfer (adjusted HR 2.76, 95% CI 2.09–3.65, *p* < 0.001), and invasive mechanical ventilation (adjusted HR 3.41, 95% CI 2.42–4.80, *p* < 0.001). Among hospital survivors, SAP was independently associated with prolonged length of stay (adjusted RoM 1.34, 95% CI 1.26–1.43, *p* < 0.001) and poor functional outcome (adjusted OR 1.87, 95% CI 1.40–2.50, *p* < 0.001).

**Table 5:** Sensitivity analyses for the association of stroke-associated pneumonia with in-hospital outcomes.

Outcome	*n*	Adjusted effect (95% CI)	*p*-value
Complete-case analysis			
In-hospital death	3,236	HR 2.29 (1.66–3.15)	<0.001
ICU transfer	3,236	HR 2.76 (2.09–3.65)	<0.001
Invasive mechanical ventilation	3,236	HR 3.41 (2.42–4.80)	<0.001
Length of hospital stay	3,038	RoM 1.34 (1.26–1.43)	<0.001
Poor outcome at discharge (mRS 3–5)	3,038	OR 1.87 (1.40–2.50)	<0.001
Day-3 landmark analysis			
In-hospital death	3,194	HR 2.18 (1.54–3.09)	<0.001
ICU transfer	3,138	HR 2.63 (1.97–3.50)	<0.001
Invasive mechanical ventilation	3,159	HR 3.12 (2.15–4.52)	<0.001

Complete-case analysis: only patients with complete data, same models as primary analysis. Landmark analysis: patients hospitalized ≥3 days; SAP within the first 3 days as a fixed exposure; follow-up from day 3. All models were adjusted for the same covariates as in Table 4. CI, confidence interval; HR, hazard ratio; ICU, intensive care unit; RoM, ratio of means; OR, odds ratio; mRS, modified Rankin Scale.

The day-3 landmark analysis, which included only patients hospitalized for at least 3 days and treated SAP occurring within the first 3 days as a fixed exposure, similarly confirmed the independent prognostic impact of SAP. Among 3,194 patients eligible for this analysis, SAP remained significantly associated with in-hospital death (adjusted HR 2.18, 95% CI 1.54–3.09, *p* < 0.001), ICU transfer (adjusted HR 2.63, 95% CI 1.97–3.50, *p* < 0.001), and invasive mechanical ventilation (adjusted HR 3.12, 95% CI 2.15–4.52, *p* < 0.001).

## 4 Discussion

In this large single-center retrospective cohort study of 3,347 patients with acute ischemic stroke, we observed that stroke-associated pneumonia occurred in 12.8% of hospitalized patients, with the majority of events (55.1%) developing within the first 72 h after admission and peak incidence on day 3. Patients who developed SAP exhibited a distinct high-risk clinical phenotype characterized by advanced age, poorer pre-stroke functional status, greater comorbidity burden, more severe neurological deficits, and higher prevalence of dysphagia and early nasogastric tube insertion. Multivariable Cox regression analysis identified older age, pre-stroke mRS score 3–5, chronic obstructive pulmonary disease, cardioembolic stroke subtype, dysphagia, and nasogastric tube insertion within 48 h as independent risk factors for SAP. Furthermore, admission NIHSS score, neutrophil-to-lymphocyte ratio, and serum albumin demonstrated significant nonlinear associations with SAP risk. Most importantly, using time-dependent Cox models to eliminate immortal time bias, we found that SAP was independently associated with substantially increased risks of in-hospital death, ICU transfer, invasive mechanical ventilation, prolonged length of stay, and poor functional outcome at discharge. These associations remained robust across complete-case and day-3 landmark sensitivity analyses.

The 12.8% incidence of SAP in our cohort aligns closely with the pooled estimate of 14.3% reported in the systematic review by Kishore et al. (4), though it falls at the lower end of the reported range. This estimate is also consistent with the 10%–15% range reported in large European and Asian cohorts (10, 22), although direct comparison is limited by differences in diagnostic criteria, case mix, and observation windows. For example, studies using CDC criteria have reported higher incidence estimates (up to 20%), whereas those using PISC criteria, as in our study, tend to report lower and more specific estimates. This further supports the importance of standardized diagnostic criteria for comparability across studies. This concordance is noteworthy given the substantial heterogeneity in diagnostic criteria across studies highlighted by Kishore et al. (4). Our use of the standardized Pneumonia in Stroke Consensus Group definitions (18) likely contributed to more accurate case ascertainment. It may explain why our incidence estimate aligns with meta-analytic averages despite single-center variation. The early temporal clustering of SAP observed in our study, with peak incidence on day 3, precisely mirrors the findings of De Jonge et al. (5), who demonstrated that pneumonia predominantly manifests within the first week after stroke and peaks on day 3. This consistent temporal pattern across diverse populations and healthcare settings underscores that SAP is not a random, late-occurring complication but rather a predictable early event demanding upfront preventive strategies.

The high-risk phenotype we identified is broadly consistent with the core components of established SAP prediction scores, including A2DS2 (age, atrial fibrillation, dysphagia, stroke severity, sex), ISAN (age, stroke severity, atrial fibrillation, pre-stroke disability), and PANTHERIS (age, stroke severity, chronic obstructive pulmonary disease, dysphagia) (12). Our findings externally validate the importance of these factors in a Chinese cohort. However, our study extends these observations by demonstrating the independent contribution of cardioembolism and the nonlinear nature of NIHSS, NLR, and albumin, suggesting that future risk models could be refined by incorporating these as non-linear terms or using different transformations. The high-risk phenotype we identified integrates multiple interconnected domains. Advanced age and pre-stroke disability (7) reflect diminished physiological reserve and reduced capacity to withstand infectious challenges. The independent association of chronic obstructive pulmonary disease is likely mediated by impaired mucociliary clearance, weakened cough mechanics, and compromised baseline respiratory status (10). The strong independent associations of dysphagia (HR 2.02) and early nasogastric tube insertion (HR 2.31) with SAP align with the classic pathogenetic pathway of impaired airway protection, silent aspiration, and subsequent pulmonary infection (9, 23). However, because nasogastric tube insertion is not a randomized intervention but a clinical decision driven by dysphagia severity and neurological deficit, this association should not be interpreted as causal. Rather, nasogastric tube insertion likely represents both a marker of more severe illness and a potential mediator of aspiration risk, and its clinical significance lies in strengthening indication assessment, timing of feeding, and standardization of care. The cardioembolic stroke subtype remained independently associated with SAP after comprehensive adjustment, extending findings from previous meta-analyses (22). This association likely reflects the tendency of cardioembolic strokes to produce larger infarct volumes and more severe neurological deficits, often involving cortical swallowing centers (7), rather than a direct pathogenic effect of the embolic mechanism itself.

A novel contribution of our study is the demonstration of nonlinear relationships between three continuous variables and SAP risk. The NIHSS admission score exhibited a progressively steeper risk gradient at higher values, suggesting that each incremental increase in NIHSS confers a greater relative risk as stroke severity worsens. The neutrophil-to-lymphocyte ratio, an established marker of systemic inflammation and stroke-induced immunodepression (6, 17), showed an accelerated risk at higher levels, supporting the concept that inflammatory burden contributes to infection susceptibility in a nonlinear, threshold-dependent manner. Conversely, serum albumin demonstrated a nonlinear protective effect, with risk escalating sharply below approximately 35 g/L. This finding extends the work of Yuan et al. (11) by revealing that nutritional status influences SAP risk not as a linear gradient but rather with a critical threshold below which vulnerability markedly increases. These nonlinear relationships have important clinical implications: they suggest that risk stratification based on simple linear assumptions may misclassify patients, and that interventions targeting these modifiable factors may yield the greatest benefit when directed at individuals who exceed or fall below specific thresholds.

Our finding that SAP independently predicts adverse in-hospital outcomes, even after rigorous adjustment for confounders and time-dependent analysis, addresses a critical methodological gap in the literature. The attenuation of crude effect estimates after multivariable adjustment (e.g., HR for death from 4.68 to 2.34) confirms that SAP is partly a marker of severe underlying stroke, as noted by Gittins et al. (6). However, the persistence of significant independent associations across all outcomes demonstrates that SAP exerts effects beyond mere reflection of illness severity. The adjusted HR of 2.34 for in-hospital death aligns with estimates from Teh et al. (19) and Rabaneda-Lombarte et al. (16), who reported similarly elevated mortality risks after accounting for confounders. A recent meta-analysis by Weng and Shen (24) reported a pooled odds ratio of 2.5 for mortality associated with SAP, which is consistent with our findings. Similarly, the 35% relative increase in length of stay observed in our study is comparable to the 30%–40% increase reported in previous cohort studies. However, many of these earlier studies treated SAP as a fixed baseline exposure, which may have overestimated the true effect. By using time-dependent models, our estimates are more conservative and likely closer to the true independent effect of SAP. The strong association with ICU transfer (HR 2.81) and invasive mechanical ventilation (HR 3.47) likely reflect the clinical cascade in which pneumonia precipitates respiratory deterioration, hemodynamic instability, and the need for escalated care (24). Among hospital survivors, the 35% relative increase in length of stay and nearly twofold higher odds of poor functional outcome underscore the substantial burden SAP imposes on patients and healthcare systems alike.

The use of time-dependent Cox models represents a significant methodological strength of our study. As Hazard et al. (14) emphasized, treating time-varying exposures, such as SAP, as fixed baseline covariates introduces immortal-time bias and overestimates prognostic effects. By modeling SAP as a time-dependent covariate, we obtained more conservative and valid effect estimates. The consistency of findings across complete-case analysis and day-3 landmark analysis further supports the robustness of our conclusions. This analytical approach addresses concerns raised by Gong et al. (13) regarding methodological limitations in previous SAP prognostic studies. It provides a template for future investigations of time-dependent complications in acute stroke.

Our findings carry several clinical implications. First, the early peak of SAP demands that preventive interventions be initiated immediately upon admission rather than deferred. Swallowing screening should be performed within the first 24 h, as recommended by Liu et al. (25), with appropriate dietary modifications and feeding pathway decisions implemented without delay. Second, the independent association of nasogastric tube insertion with SAP, even after adjustment for dysphagia, suggests that while tube feeding may be necessary, it should be accompanied by rigorous protocols to minimize reflux and microaspiration (26). Third, the nonlinear relationships of NIHSS, NLR, and albumin with SAP risk suggest that patients exceeding certain thresholds warrant heightened surveillance and potentially targeted interventions such as early nutritional support for those with hypoalbuminemia. Fourth, the independent prognostic impact of SAP supports the development and implementation of care bundles incorporating swallowing management, oral care, head positioning, and early mobilization, which have demonstrated efficacy in reducing SAP incidence (20).

### 4.1 Limitations and future directions

Several limitations warrant consideration when interpreting our findings. First, as a single-center, retrospective study conducted in a tertiary hospital neurology department, our cohort may not be representative of patients managed in community hospitals or in other healthcare systems. Referral patterns and admission practices may have influenced case mix, potentially limiting generalizability (2). Second, despite using standardized consensus criteria and independent adjudication, SAP identification remained dependent on documentation quality in medical records. Variations in symptom recording, timing of chest imaging, and clinical suspicion may have introduced information bias, though the low missing-data rates mitigate this concern to some extent. Third, although we adjusted for an extensive set of confounders, residual confounding cannot be ruled out. We were unable to account for potentially important variables, including precise infarct volume and location, intensity of oral care, sedative medication use, and early rehabilitation interventions. Additionally, VAP was not analyzed separately from SAP, which may have introduced overlap in patients requiring invasive mechanical ventilation; future studies should distinguish these entities to clarify their distinct mechanisms and prognostic implications. Antibiotic use was also not recorded as a structured variable and was initiated after SAP diagnosis; therefore, it was not included as a covariate to avoid overadjustment bias, as it lies on the causal pathway between SAP and outcomes. Future prospective studies should standardize antibiotic data collection, including agent, timing, and duration. Fourth, our outcome assessment was limited to the in-hospital period, precluding evaluation of SAP’s impact on mid-term and long-term outcomes such as 30-day or 90-day mortality and functional recovery (19, 24).

These limitations highlight key directions for future research. Prospective multicenter studies with standardized SAP surveillance are needed to validate our findings and clarify the relationship between lesion topography and SAP risk (7). The nonlinear relationships we identified warrant confirmation, and follow-up should extend beyond discharge to determine whether SAP’s prognostic impact persists. Randomized trials should evaluate whether targeting modifiable factors—particularly nutritional optimization in hypoalbuminemia and airway protection in high NLR—can reduce SAP incidence. Despite these limitations, the large sample size, rigorous adjudication, comprehensive adjustment, time-dependent analysis, and consistent sensitivity results support the internal validity of our conclusions.

## 5 Conclusion

Stroke-associated pneumonia occurred in 12.8% of hospitalized patients with acute ischemic stroke in this large cohort, with the majority of cases developing within the first 72 h after admission and peaking on day 3. Older age, pre-stroke disability, chronic obstructive pulmonary disease, cardioembolic stroke, dysphagia, and early nasogastric tube insertion were independently associated with SAP risk. Admission NIHSS score, neutrophil-to-lymphocyte ratio, and serum albumin exhibited nonlinear relationships with SAP, revealing threshold effects with clinical relevance. Using time-dependent models that eliminated immortal time bias, SAP remained independently associated with substantially increased risks of in-hospital death, ICU transfer, invasive mechanical ventilation, prolonged hospitalization, and poor functional outcome at discharge. These findings underscore that SAP is not merely a marker of severe stroke but an independent contributor to adverse in-hospital trajectories. The early temporal window for SAP occurrence demands forward-shifted preventive strategies, including immediate swallowing assessment, judicious selection of feeding pathways, airway protection protocols, and nutritional optimization in high-risk patients during the critical first days of hospitalization.

## Data Availability

The original contributions presented in the study are included in the article/supplementary material, further inquiries can be directed to the corresponding author.
